# Cobalamin and Folic Acid Status in Relation to the Etiopathogenesis of Pancytopenia in Adults at a Tertiary Care Centre in North India

**DOI:** 10.1155/2012/707402

**Published:** 2012-04-01

**Authors:** M. Premkumar, N. Gupta, T. Singh, T. Velpandian

**Affiliations:** ^1^Department of Medicine, Maulana Azad Medical College and Associated Hospitals, Bahadur Shah Zafar Marg, New Delhi 110002, India; ^2^Department of Pathology, Maulana Azad Medical College and Associated Hospitals, Bahadur Shah Zafar Marg, New Delhi 110002, India; ^3^Department of Ocular Pharmacology, Dr. Rajendra Prasad Eye Centre, All India Institute of Medical Sciences, Ansari Nagar, New Delhi, India

## Abstract

*Background*. Pancytopenia has multiple etiologies like megaloblastic anemia, aplastic anemia, leukemia, and various infections. We investigated the clinical, etiological and hematological profile including bone marrow morphology of patients with pancytopenia in relation to their vitamin B12 and folic acid status at a tertiary care referral hospital in north India. *Methods*. A total of 140 consecutive patients with pancytopenia were selected from June 2007 to December 2008. Bone marrow examination and other tests were carried out as warranted, including serum cobalamin and folate assays using liquid chromatography mass spectroscopy (LC MS/MS). *Results*. The study population consisted of 92 males and 48 females with a mean age of 32.8 years. Megaloblastic anemia 60.7%, aplastic anemia (7.8%), and leukemia (9.2%) were common causes. Infectious causes (16.4% of all cases) included leishmaniasis, HIV–AIDS, malaria and tuberculosis. Severe cobalamin deficiency (B12 < 100 pg/mL) was seen in 81% of all patients including 91.6% of patients with MA. In contrast, only 7.14% of all pancytopenic patients were folate deficient. Folate deficiency (<5 ng/mL) was seen in just 5% MA patients. Combined cobalamin and folate deficiency was seen in 5 patients (3.51%). *Conclusion*. Cobalamin deficiency was found to be more common in our setting and is largely underdiagnosed in the age of folate supplementation. Infectious diseases like tuberculosis, leishmaniasis, and increasingly HIV are important and treatable causes of pancytopenia. This is in contrast with the developed nations where the bulk of disease is due to malignancy or marrow aplasia.

## 1. Introduction

Pancytopenia is diagnosed when there is a reduction in all three hematopoetic cell lines. This is seen as reduction in the white cell count, hemoglobin, and platelet count which is most often the result of bone marrow infiltration or failure, anticancer chemotherapy, hypersplenism, systemic diseases, and infections like HIV, tuberculosis, leishmaniasis, and so forth [1, 2]. While there are several published studies on the hematological diagnosis of pancytopenia on basis of bone marrow morphology, few have attempted to explore the underlying etiology and clinical course of the disorders leading to this condition. In this study, we hoped to evaluate the demographic, nutritional, socioeconomic and age patterns of the myriad disorders which cause pancytopenia and develop an approach to the diagnosis of this condition in our patient population.

## 2. Materials and Methods

This prospective study comprised of adults drawn from the outpatient/inpatient/emergency/referral services of Maulana Azad Medical College, Lok Nayak Hospital and other associated hospitals, a tertiary care referral centre in New Delhi, north India. The period of observation was from June 2007 to December 2008. Overall, 160 consecutive patients were recruited in the study with written informed consent. Of these, 20 patients were excluded due to one or more exclusion criteria. The inclusion criteria for the study were

1. age > 18 years of either sex,
2. pancytopenia as defined by the presence of the following:
  - Hb < 10 gm/dL in female subjects and <11 gm/dL in male subjects,
  - Total leukocyte count < 4 × 10^9^ cells/L,
  - Platelet count < 150 × 10^9^/L.

The exclusion criteria for this study were

1. cancer chemotherapy recipients or concomitant use of colony stimulating factors,
2. recipients of multiple blood/blood component transfusions prior to presentation in our hospital,
3. patients who were too sick to undergo the proposed diagnostic workup.

### 2.1. Study Design

A questionnaire was used to document demographic data, clinical presentation, dietary history, past history of anemia, blood transfusions and drugs, administration of hematinics, and blood transfusions including transfusion of blood components and radiation exposure. This was followed by a detailed clinical examination, particularly making note of pallor, jaundice, hepatomegaly, splenomegaly and lymphadenopathy and other features peculiar to conditions like B12-deficient anemia like hyperpigmentation of skin, knuckles, and so forth. Patient outcome and improvement with followup was also documented for a period of at least four months after discharge. The laboratory tests performed were as follows.

Complete blood count (CBC) with a differential count and red blood cell indices was estimated using the automatic cell counter MS 9 Hematology Analyzer, (Melet Schloesing Laboratories, USA). A blood film was stained by the Wright stain and evaluated for red cell morphology, band forms, platelet morphology and number, and any atypical cells/parasite. Reticulocyte count using 1% brilliant cresyl blue for supravital staining was done. Liver function tests, liver enzyme levels, renal function tests, serum ferritin and lactate dehydrogenase (LDH) levels were also done in all patients.

**Bone Marrow Examination**

Bone marrow aspiration and wherever required, a trephine biopsy was performed. Biopsy was required in case of dry tap, marrow fibrosis, inadequate first aspirate, marrow aplasia, and hematological neoplasms. Further tests were carried out as warranted by the clinical context and the results of baseline investigations.

**Vitamin B12 and Folate Assays**

Blood was collected in vacutainers with aseptic technique, centrifuged immediately and serum samples were stored at −70°C till assay was done. The HPLC (high pressure liquid chromatography) standard reagent for folic acid was procured from Qualigens Pharmaceuticals and cyanocobalamin from Khemi Labs. The samples were processed to get serum and solid phase extraction was done by mixing 500 *μ*L of sample with 10 *μ*L of internal standard (sildenafil) and processing with solvent acetonitrile and distilled water through solid phase extraction cartridges to procure the test samples for liquid chromatography coupled to isotope dilution tandem mass spectroscopy (LC-MS/MS).

The normal range of cobalamin was 200–700 pg/mL and folate was 5.0–15 ng/mL. The method was calibrated using 20 control samples of asymptomatic individuals without anemia. The normal range of vitamin B12 and folic acid depends on the kind of assay used. Conventionally B12 estimation was done by microbiological assays, ELISAs (enzyme linked immunosorbent assay), HPLC, or radioimmunoassay (RIA). We used LC MS/MS for estimation of both B12 and folate, which is now a candidate reference method for vitamin estimation with good reproducibility and reduced interlaboratory and intermethodology variation [3, 4]. Other studies have reported the limits of detection of folic acid in serum as 0.07 nmol/L. Coefficient of variation for within and between run imprecision was <10% for folate concentration >2 nmol/L. Total folate concentration determined by RIA was 9% lower than results obtained with LC-MS/MS [5]. Another study for B12 estimation using LC-MS/MS demonstrated specificity by the retention characteristics and peak purity with the vitamin B12 standard. The calibration graph plotted with six concentrations of vitamin B12 showed a regression coefficient *R* > 0.9997. The relative standard deviation was below 3.2% [6]. Homocysteine was estimated in serum by conventional Enzyme Immunoassay (EIA) method using commercially available kits (Bio-Rad Laboratories Incorporated, India).

### 2.2. Statistics

The measurements obtained by the above-mentioned studies were used to calculate mean values for each patient and all cases as a whole. The Students' *t*-test was used for both independent variables. The paired sample *t*-test was performed to compare the difference of means between groups. *P* < 0.05 was considered significant. Data was analysed with SPSS software version 13 (SPSS, Chicago, IL, USA).

## 3. Observations and Results

The study population consisted of 140 patients with a mean age of 32.8 years with range of 18–80 years. There were 92 males and 48 females in the study group (M : F ratio 1.9 : 1). More than half (59%) of all patients belonged to a younger age group (18–30 years) including 56% of all females and 55% of all males. Most of the patients (76.4%) belonged to a lower socioeconomic status based on occupation, income, and education. In our study, MA was the largest group comprising 60.7% of all cases. Aplastic anemia accounted for 7.8% while leukemia (including acute myeloid leukemia, acute non myelogenous leukemia, undifferentiated leukemias, chronic myeloid leukemia in blast crisis, and one case of lymphoma) accounted for 9.2% cases, respectively (see Table 1).

**Figure 1:**
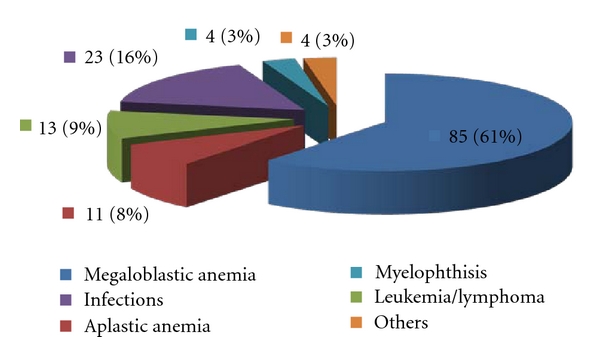
Clinicopathological diagnostic groups in pancytopenia.

**Figure 2:**
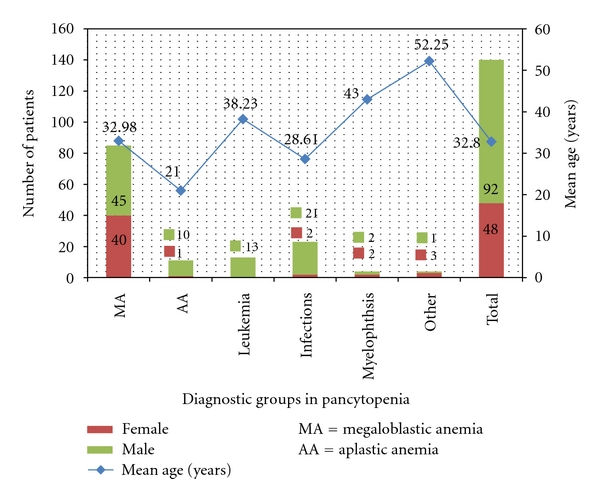
Age and gender distribution across diagnostic groups in pancytopenia.

Infectious causes accounted for 16.4% of all cases including cases of kala azar (8), HIV-AIDS (8), chronic malaria (4), and disseminated tuberculosis (3). There were two cases of storage disorder including one case of Gaucher's disease and two cases of metastatic carcinoma. We also encountered one case of myelodysplastic syndrome in our study. In the older patient subgroup aged 31–80 years, there were 39 (75%) patients with MA, 4 (7.7%) with aplastic anemia, and 6 (11.5%) with infectious causes of pancytopenia. Aplastic anemia was seen in patients in their 2nd or 3rd decade. They were younger as compared to pancytopenics in the other diagnostic groups (*P* = 0.001) (see Figures 1 and 2).

All patients were symptomatic, the common symptoms being easy fatigability (98%), weight loss (66%), fever (67%), abdominal fullness (72%), early satiety after meals (80%), pain abdomen (66%), swelling of feet (26%), and shortness of breath (27%). Anemia was severe enough to present as pallor in all patients. About 17% of all patients had icterus including 21.2% of patients with MA. In the group with MA, 10.6% of patients had hyperpigmentation of skin while 18.8% of all patients had knuckle hyperpigmentation, which was found to correlate significantly with this diagnosis (*P* < 0.005). Glossitis (26%), angular cheilitis (13%), and pedal edema (11%) were common in pancytopenia. Glossitis was present in 36% of patients with MA. The largest splenic enlargement was encountered in the category of infections especially kala azar and the storage disorders. Another significant finding was that 16% of all pancytopenic patients had no splenomegaly including 7% of MA and 23% of leukemia patients.

One fourth of pancytopenia had a clinical bleed and a quarter of them had bleeding from more than one site. Half of the bleeding cases, that is, 18/35 (51%), occurred at a platelet count of 50–100 × 10^9^/L. The most common sites of bleeding were bleeding per rectum and menorrhagia each affecting 11/140 (7.9%) patients. Epistaxis was a significant finding in patients of aplastic anemia affecting 18% of these cases. Up to 25% of women with MA had menorrhagia. Skin bleeding (3.5%) and easy bruisability (4.7%) were also noticed in the MA group. Up to 30.8% of patients with leukemia had significant postintervention bleeding from central line sites or after marrow aspiration.

### 3.1. Hematological Parameters in Pancytopenia

Some interesting correlates could be assessed from this study. The mean hemoglobin level in all patients of pancytopenia was 5.3 ± 1.6 g/dL. Mean Hb was just 3.8 g/dL in the group of aplastic anemia. Severe anemia (Hb < 5 g/dL) was seen in 40% of MA, 72% of aplastic anemia, and 46% of patients with leukemia. Aplastic anemia patients also had a significantly lower mean platelet count (42 × 10^9^/L) as compared with other pancytopenic patients (82 × 10^9^/L). A platelet count of <40 × 10^9^/L correlated with a diagnosis of aplastic anemia (*P* = 0.001). Likewise the absolute neutrophil count was also markedly reduced in this group. The mean reticulocyte count in this group was 0.71 as compared with 1.76 for other pancytopenics. Macrocytosis (MCV ≥ 100 fl) was seen in 33% and macroovalocytosis in another 34% of MA. An MCV of >95 fl and an RDW ≥ 16 each correlated with a diagnosis of MA (*P* = 0.001). Hypersegmentation (≥5 nuclear lobes in neutrophils) was seen in 34% of MA and was a specific finding.

### 3.2. Role of Bone Marrow Examination

Bone marrow examination corresponded with the clinical diagnosis in 126/140 patients, that is, almost 90%. Bone marrow aspirate was done in all 140 cases and aspirate with biopsy was performed in 130 cases. The reporting pathologists were blinded to the results of the vitamin assays and were provided with clinical data and hematology reports. The diagnostic classification for haematological diseases by the World Health Organization was followed in all cases. We found bone marrow aspirate alone corresponded with the diagnosis in only 98/140 (70%) patients while biopsy allowed better assessment of cellularity, fibrosis, and extent of bone marrow involvement, in addition to diagnosing 126/140 (90%) patients. The cellularity was increased in 100/140 (71%), decreased in 25/140 (17%), and was normal in 15/140 (10%) cases. The marrow was hypocellular in all cases of aplastic anemia, 25% of leukemia patients, and up to 26% of patients with infections. Ineffective erythropoesis was seen in megaloblastic anemia; erythroid hyperplasia was reported in 60% of patients with 49% showing mild and 29% showing moderate-to-severe dyserythropoesis. The erythroid reaction was macronormoblastic in 29% and megaloblastic in 50% cases in this group. Dysmyelopoesis was seen in 34% of MA patients. Bone marrow infiltration was seen in leukemia and lymphoma in which blast cells replace the hematopoetic precursors. We also encountered two cases of metastatic cancer-gastric signet cell cancer, and anaplastic carcinoma with marrow involvement. Myelodysplastic syndrome was detected in one patient but no dysmyelopoesis was seen in the marrow. Bone marrow involvement was seen in all the three patients of disseminated tuberculosis (TB) who presented with pancytopenia. In one of these patients, polymerase chain reaction (PCR) on marrow aspirate for TB was positive on the bone marrow aspirate and while granulomas with caseation were seen in all three. Storage disorders exhibit bone marrow infiltration and hypersplenism due to marked splenomegaly. Hypersplenism was seen with visceral leishmaniasis, chronic malaria, and with chronic liver disease with portal hypertension. Decreased iron stores were seen in 15% of cases with megaloblastic anemia. Bone marrow iron stores were comparable with serum ferritin in cases of megaloblastic anemia, aplastic anemia, and myelophthisis. However, serum ferritin was found to be elevated in cases of leukemia and infections, especially cases of sepsis and did not always correlate with stained marrow iron status so results need to be interpreted carefully.

### 3.3. Biochemical Tests

Total bilirubin was elevated in 25% of all patients while 17% of pancytopenic patients had icterus. Of the 20 patients with indirect hyperbilirubinemia, 12 (60% of all) belonged to MA group (*P* = 0.01). Total protein was low in just three patients. Hypoalbuminemia was common in the group with infections affecting nearly 35% of patients. Interestingly about 18% of patients of both MA and aplastic anemia had hypoalbuminemia. Hypergammaglobulinemia (>4.2 g/dL) was not found in any patient in this study. LDH was elevated in up to 46% of all patients including 40% of MA and 46% of leukemias. This parameter has been found to correlate with disease and response to therapy in other studies. An elevated LDH level (>250 IU/L) significantly correlated with a diagnosis of MA as compared with nonmegaloblastic pancytopenia (rank correlation *r* = 0.91) [7, 8].

### 3.4. Vitamin Assays in Pancytopenia

**Figure 3:**
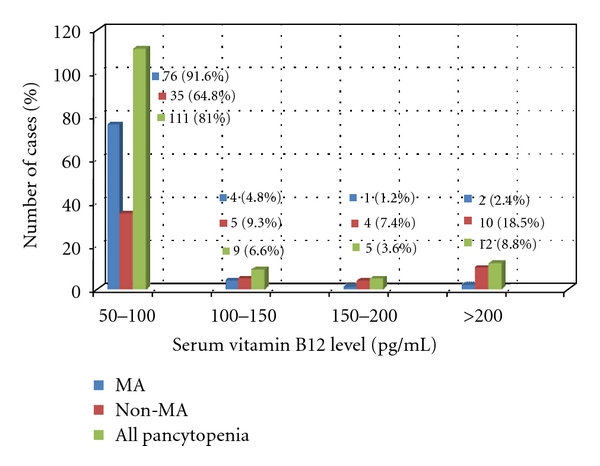
Severity of Vitamin B12 deficiency in pancytopenia.

**Figure 4:**
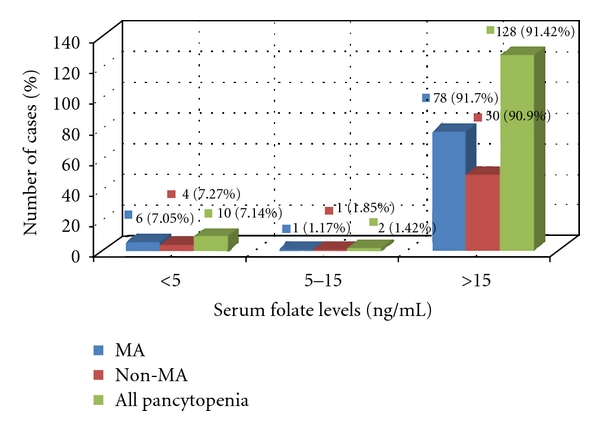
Severity of Folate deficiency in pancytopenia.

On evaluating the vitamin levels in patients of MA versus other pancytopenic patients, B12 levels were statistically lower and homocysteine levels statistically elevated in MA (*P* < 0.001) (See Table 3). The mean levels of folic acid and ferritin were comparable in both groups. We noted that patients with bleeding manifestations had a lower serum ferritin. Iron deficiency as defined by serum ferritin <12 ng/mL or depleted bone marrow iron was found in 17.14% cases. Mean ferritin levels in patients with bleeding (32.3 ± 18.4 ng/mL) was found to be lower than in those without (42.9 ± 34.9 ng/mL) but the difference was not statistically significant. It was found that 81% of all patients had severe B12 deficiency (B12 < 100 pg/mL) including 91.6% of patients with MA. In contrast to this, only 7.14% of all patients were folate deficient. Folate deficiency <5 ng/mL was seen in just 7% MA patients (see Figures 3 and 4).

### 3.5. Dietary Associations of Pancytopenia

The majority (71.4%) of patients were lactovegetarian. There were no vegans in this study and very few patients with frequent nonvegetarian intake. The lactovovegetarian (12.9%) cases behaved similar to the infrequently nonvegetarian group. Only 15.7% of patients consumed fish, meat, and poultry. On comparing the hematological parameters with diet, no significant difference was found in the mean Hb, TLC, and platelet count levels in the lactovegetarians and nonvegetarian subgroups. The RDW, S. ferritin and RBC indices in both groups were comparable. The mean B12 level in all cases of pancytopenia was 157.6 pg/mL (normal range 200–700 pg/mL) and though mean B12 levels were found to be higher in the non vegetarian population (169.3 pg/mL) as compared with vegetarian group (129.5 pg/mL), this difference was not statistically significant and both groups were found to have cobalamin deficiency. The folate and LDH levels were, however, significantly different between the vegetarian and nonvegetarian folate levels being lower and LDH being higher in the vegetarian group. Another significant finding was 25.7% of all patients had received proton pump inhibitor therapy (PPI). This included 23.5% patients with megaloblastic anemia, 26% of leukemia patients, and 26% of patients with splenomegaly. However though the MCV was higher and vitamin B12 levels lower in the group consuming PPI, the difference was not statistically significant. Also, 24.3% of patients consumed tobacco in some form including 23% of patients with leukemia and 7.1% patients consumed alcohol.

### 3.6. Neurological Features

In this series of 140 patients, 26.4% of all patients had neurological and/or psychiatric complaints including symptoms of depression, memory loss and irritability, which included 22% of all MA patients. Notably, up to 40% of patients with leukemia had concomitant depression at some point during the course of illness requiring psychiatric referral and treatment. Subjective motor weakness (7%) and gait ataxia (12%) were reported by patients with MA. Sensory deficits of impaired vibration sensation and joint position sense were found in 8.2% cases. Objective motor weakness was found in 7.1% of these patients. These subjects underwent nerve conduction studies (NCS) and 8 cases of demyelinating peripheral neuropathy were detected. Most subjects with B12-deficient MA reported a subjective improvement in their symptoms of memory loss and mood after cobalamin therapy. There was complete or partial recovery in the motor and sensory neuropathic complaints at 3 months after therapy in subjects with cobalamin deficiency, with conversion to a normal NCS in 3 cases where the test was repeated.

### 3.7. Other Tests

Splenic aspirates were performed in two patients of visceral leishmaniasis whose marrow aspirates were negative, which showed the characteristic LD (leishmania donovani) bodies. Diagnostic lymph node biopsy was done in four cases of HIV AIDS with disseminated tuberculosis, all of which showed caseation and poorly formed granulomas; and in one case of non Hodgkin's lymphoma. All patients of aplastic anemia in this study tested negative for hepatitis B and C and HIV. Parvovirus B19 serology was done in five patients but was negative in all of them.

### 3.8. Patient Outcome and Followup

Eighty-six (61.4%) of the patients reported back to the outpatient department after discharge including 55/85 (64.7%) with MA who were on injectable B12 therapy, 6/13 (46.2%) of leukemia who were on chemotherapy, 14/23 (60.8%) with infections, 8/11 (71.1%) with aplastic anemia, and 2/4 (50%) with myelophthisis. Eight patients had expired in the first admission itself including three cases of leukemia, both cases of metastatic carcinoma, two cases of HIV AIDS with tuberculosis, and one case of chronic malaria. Of the fifty-five cases with MA, only forty reported regularly for followup at the end of one year and had a good symptomatic and hematological recovery. On examination of these patients, all but two patients showed regression of splenomegaly by three months. Knuckle hyperpigmentation was found in just 8.3% of MA patients on followup as compared to 18.8% patients at presentation.

Eight cases of acute leukemia received induction chemotherapy and six achieved remission. However, only four of these patients survived at the end of two years; of which two had suffered a relapse and were referred for bone marrow transplantation (BMT). Of the eleven cases of aplastic anemia, nine were managed with immunosuppressants or antithymocyte globulin and supportive therapy, and all survived at the end of one year. Two patients were referred for BMT. Of the surviving six cases with HIV AIDS, two expired at the end of one year while the others achieved hematological recovery and were on regular followup. All eight cases of leishmaniasis received amphotericin B therapy and recovered completely. All three cases of disseminated tuberculosis were managed with ATT (antitubercular therapy) but only two were on followup after four months. Subjects with vitamin deficiency were treated with oral and/or injectable cobalamin and folate therapy in addition to therapy for the primary disease.

Table 4 on the hematological aspects on followup shows significant changes in Hb, TLC, platelet count, MCV, MCH and reticulocyte count when compared with Table 2 which shows the same parameters at the time of presentation. The mean Hb improved to 12.3 ± 0.97 g/dL in MA and to 9.4 ± 2.0 g/dL in AA. The mean RDW was 16 at presentation which was corrected with therapy to a mean value of 12.82 ± 1.11 in MA. Likewise mean LDH was 604.8 ± 618 IU/L at the time of presentation which improved to 89.0 ± 33.7 IU/L at followup in MA corroborating earlier studies stating that LDH may be used as a marker of response to therapy.

## 4. Discussion

Six broad diagnostic groups could be identified in pancytopenia. Megaloblastic anemia constituted the largest group comprising 60.7% of all cases. Aplastic anemia (7.8%), leukemia (9.2%), and infections (16.4%) were the next most common causes.

### 4.1. Other Studies in India

A number of studies have been done on pancytopenia in India documenting the geographic and demographic trends of this diverse disease process. In a study at Chandigarh, the most common cause of pancytopenia as revealed by bone marrow examination was aplastic anemia (40.6%) followed by megaloblastic anemia (23.26%) [9]. Another study at New Delhi found megaloblastic anemia, aplastic anemia, leukemia, myelodysplastic syndrome, paroxysmal nocturnal hemoglobinuria, overwhelming viral infections, and drug-induced pancytopenia as the most commonly diagnosed causes of pancytopenia [10]. In contrast a recent study from eastern India has reported aplastic anemia (20.72%) as the commonest cause, followed by hypersplenism due to chronic liver disease (11.71%) and leishmaniasis (9%) [11] (see Table 5).

These differences in the etiology of pancytopenia are due to differences in population characteristics such as, age patterns, nutritional status, socioeconomic parameters and the prevalence of infections in a geographic region [9–13]. Since our study excluded patients aged less than 18 years, the number of aplastic anemia cases was reduced as a large proportion of this disease affects patients in the second decade which would be considered in the pediatric category. This correlates with the finding that studies with younger subjects have reported aplastic anemia more commonly. Similarly other studies included recipients of cytotoxic drugs and chemotherapy and therefore reported marrow hypoplasia as being common. This group was specifically excluded in our study as the etiology of marrow suppression due to primary disease or chemotherapy is unclear in such cases. Also, our patient population was mainly of the lower socioeconomic status, and the cobalamin deficiency may be attributed to an inadequate diet. This fact partially explains the higher proportion of MA cases in the present study. We were also stringent in the application of the exclusion criteria and cases who received blood transfusions and injectable drug therapy elsewhere were not included as these would have affected vitamin assays and marrow morphology. 

Ours being a referral tertiary care hospital, we saw cases from all over north India. The prevalence of infections in a community such as malaria, kala azar, HIV AIDS, and enteric fever varies from region to region. Of the eight patients with HIV, seven had concomitant tuberculosis. While HIV per se can cause myelosuppression, the presence of TB in these patients may have added further to the marrow dysfunction resulting in pancytopenia. More cases of HIV AIDS with pancytopenia were encountered but they were excluded as many had received blood transfusions prior to referral or were too ill for the proposed work up.

### 4.2. Studies in Other Countries on Pancytopenia

A number of studies have been done in other countries reflecting the importance of socioeconomic and geographic parameters on the etiology of this condition. Infections such as tuberculosis, HIV, and vitamin deficiencies are treatable and frequent causes of pancytopenia in developing countries. This contrasts with studies in the developed world where the bulk of disease is due to malignancy or marrow aplasia [15–21]. A study in Zimbabwe concluded that megaloblastic anemia, aplastic anemia, and AIDS are the most common causes of pancytopenia [16]. Aplasia was the most frequent cause of severe pancytopenia. In a study done in Pakistan in 2001, megaloblastic anemia, hypersplenism and aplastic anemia were the main causes of pancytopenia [17]. In contrast, a French review of 213 consecutive adult pancytopenics showed malignant myeloid disorders (acute myeloid leukemias, MDS, and myelofibrosis) represented 42% and various malignant lymphoid disorders 18% of the cases. Aplastic anemia (10%), vitamin deficiencies (7%) and nonhematological pathology (10%) were other causes [18].

*Bone marrow examination * concurred with the clinical diagnosis in 126/140 patients, that is, almost 90 percent. In some cases, when the first bone marrow aspirate was not diagnostic or showed hemophagocytic syndrome, a second aspirate and biopsy was performed and more often than not clinched the diagnosis. This was especially so with cases of focal deposit disorders like lymphoma and metastatic carcinoma, and infections like kala-azar, tuberculosis, and chronic malaria. We also noted two cases of aleukemic leukemia and a case of lymphoma which were masquerading as refractory MA on basis of vitamin B12 assays and bone marrow aspirates. These three cases were however excluded from the study because they received prior treatment including blood transfusions elsewhere before referral to our hospital. This highlights the necessity of a good specimen of aspirate and biopsy in marrow examination in refractory MA or pancytopenia.

### 4.3. Vitamin Assays

Severe cobalamin deficiency (serum B12 < 100 pg/mL) was seen in 81% of all patients including 91.6% of patients with MA, the lowest level being 53 pg/mL. Only 12% of patients had levels above 200 pg/mL. Vitamin B12 levels were significantly lower in MA as compared with the other groups. In contrast to this, only 7.1% of all pancytopenic patients were folate deficient. Folate deficiency <5 ng/mL was seen in just 5% MA patients. Combined deficiency of cobalamin and folate was seen in just 5 patients, that is, (3.5%). The mean levels of serum folate did not significantly differ between the diagnostic groups and also there was no statistical difference between folate levels in megaloblastic versus nonmegaloblastic pancytopenic patients. The issue of the low frequency of folate deficiency in this study can be explained by various factors. Firstly, recipients of blood transfusion and injectable drug therapy were excluded as these would have affected vitamin assays. However, oral folic acid or multivitamin supplementation prior to presentation at our centre may have resulted in normal folate levels as even a single folate-rich meal can change serum concentration [22]. Secondly, cobalamin and folate measurements in serum show wide intermethodology variability partly due to usage of inadequate standards and different techniques like radioimmunoassay (RIA) and microbiological methods. The World Health Organisation (WHO) Expert Committee on biological standardization established lyophilized pooled serum IS 03/178 as the first international reference standard for B12 and folate using candidate reference methods based on LC-MS/MS to reduce interlaboratory variability [23]. The RIA technique (Bio-Rad QuantaPhase II) used in the United States National Health and Nutrition Examination Surveys (NHANES) for 25 years is now being phased out to be replaced by LC-MS/MS for folate estimation [24]. Moreover serum cobalamin levels may not be indicative of actual deficiency. Cobalamin levels may be falsely high in patients with megaloblastosis due to nitrous oxide, transcobalamin II (TCII) deficiency, inborn errors in cobalamin metabolism, and myeloproliferative disorders. On the other hand, serum cobalamin levels can be falsely low within some patients with folate deficiency, individuals on high doses of ascorbic acid, pregnant women, and persons with transcobalamin I (TCI) deficiency [25, 26]. So clinicians need to interpret results carefully. 

Nonetheless, another hospital-based study done in Delhi on 107 patients of MA revealed cobalamin deficiency in 78 patients (65%), combined cobalamin and folate deficiency in 20 patients (12%), and pure folate deficiency in 8 patients (6%) indicating a similar pattern [27]. Another study on asymptomatic adults in Delhi revealed that 46.9% of “normal” subjects had subnormal levels of serum cobalamin or folate, cobalamin deficiency being five times as common as folate deficiency [28]. Further population-based studies are therefore required before conclusions are generalized. Serum homocysteine was done in only 38 patients including 26 with MA. It was elevated in all MA patients as compared with the rest and the difference was statistically significant (**P** = 0.002). Of the megaloblastic patients with hyperhomocysteinemia, 19 (73%) were vegetarian. A recent study reported *≈*75% of a selected urban population from western India had metabolic evidence (hyperhomocysteinemia and methylmalonic acidemia) consistent with cobalamin deficiency that can only partly be explained by a vegetarian diet [29]. 

In recent years, there has been a growing interest in the vitamins folic acid and cobalamin because of the realization that the status of the vitamins in populations is less than adequate, and that such inadequacy may be linked to adverse public health outcomes. This concern has prompted several countries like the United States, Canada, and so forth to fortify food grain products with folate, while additional countries are considering doing so in the near future [30]. 

### 4.4. Dietary Factors

Vegetarians comprised 71% of all patients. Though the majority of MA patients were lactovegetarians, a good 29% were consuming a nonvegetarian diet. We wish to emphasise here that B12 deficiency should not be assumed to affect vegetarians only as the frequency of consumption of nonvegetarian food intake was found to be low even in the non vegetarian group which might explain the high frequency of B12 deficiency in this study. This important facet of the north Indian dietary habit needs to be considered in any analysis of vitamin/diet deficiency while comparing with western data or with reports from the other parts of the country [31]. 

Other than a vegetarian diet, the cause of cobalamin deficiency in the subjects in the current study was not clear. Tropical sprue, giardiasis, and other gastrointestinal infections are common in India, and these may lead to malabsorptive states and cobalamin deficiency. We did perform barium studies and upper gastrointestinal endoscopy with biopsies in symptomatic patients but the yield of such tests remains low [32, 33]. Further evaluation of the cause of B12 deficiency is required in such cases including anti-intrinsic factor or antiparietal cell antibody tests and evaluation of genetic and familial disease. 

## 5. Conclusion 

**Figure 5:**
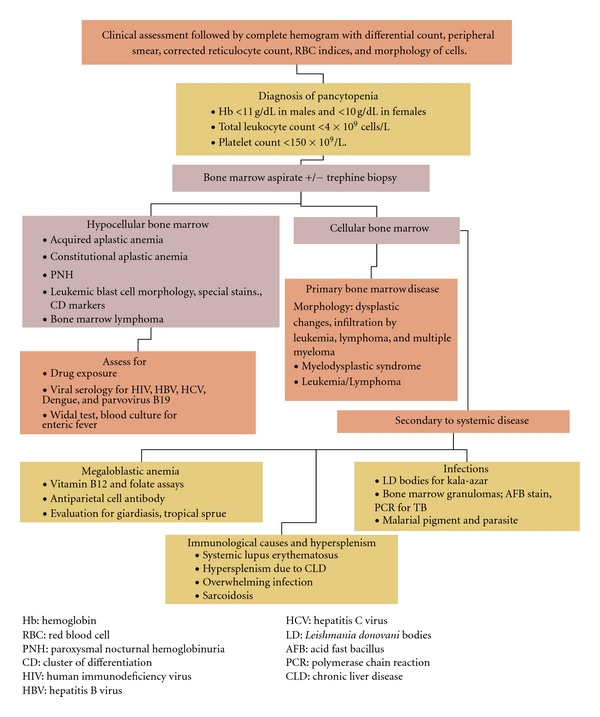
Algorithmic approach to the investigation of pancytopenia.

Pancytopenia has multiple etiologies. Data regarding pancytopenia in an adult Indian population is lacking which prompted this study. People in India and other developing nations continue to suffer from vitamin deficiencies which cause anemia and pancytopenia. These are easily preventable if diagnosed and treated early. Better health awareness will reduce the burden of disease. Infectious diseases like tuberculosis, kala-azar, and increasingly HIV are important and treatable causes. This is in contrast with the developed nations where the bulk of disease is due to malignancy or marrow aplasia. A careful and individualized approach to the diagnosis and management of this condition is needed (See Figure 5). The role of dietary deficiency of cobalamin cannot be emphasized enough, even in nonvegetarian patients as subclinical deficiency is common in the Indian population and manifests with both hematological and neurological consequences. Our study revealed that multifactorial causation for pancytopenia in our patient population is probable and occult cobalamin deficiency may contribute to the burden of hematological disease in patients with other primary diagnoses. Therefore B12 status needs to be assessed either clinically or by laboratory methods in high risk groups, and also in case of poor response to primary therapy. Cobalamin (either oral or parenteral) and folic acid therapy should be considered in addition to treatment for the primary diagnosis, depending on the clinical scenario. Concerns raised about the risks of unmasking undiagnosed B12 deficiency in the age of folate supplementation are compelling especially in the Indian population. Further recommendations regarding folate or B12 supplementation in the general population can only be made after more population-based studies are completed. 

## Figures and Tables

**Table 1:** Etiological diagnosis in pancytopenia.

S. No.	Diagnosis	Females	Males	Total	% of total
1	Megaloblastic anemia	40	45	85	60.7
2	Aplastic anemia	1	10	11	7.9
3	Acute leukemia^†^	0	11	11	7.9
4	HIV-AIDS	0	8	8	5.7
5	Kala azar	1	7	8	5.7
6	Chronic malaria	1	3	4	2.9
7	Disseminated tuberculosis	0	3	3	2.1
8	Chronic liver disease	2	0	2	1.4
9	Lymphoma	0	1	1	0.7
10	Myelodysplastic syndrome	0	1	1	0.7
11	CML with blast crisis	0	1	1	0.7
12	Metastatic anaplastic carcinoma	0	1	1	0.7
13	Metastatic gastric carcinoma (signet cell)	1	0	1	0.7
14	Niemann Picks disease	0	1	1	0.7
15	Gaucher's disease	1	0	1	0.7
16	Acute subdural hematoma with transient pancytopenia	1	0	1	0.7

Total		48	92	140	100

HIV-AIDS: human immunodeficiency virus-acquired immunodeficiency syndrome; CML: chronic myeloid leukemia.

^†^Includes 4 patients of myeloid leukemia and 4 patients of nonmyelogenous leukemia, 2 undifferentiated leukemias, and one case of acute promyelocytic leukemia.

**Table 2:** Hematological parameters in pancytopenia.

	Megaloblastic anemia	Aplastic anemia	Leukemia	Infections	Myelophthisis	Other	All pancytopenia
	Mean ± standard deviation						
Hb (g/dL)	5.2 ± 1.6	3.8 ± 1.7	5.7 ± 1.3	6.1 ± 1.3	5.0 ± 1.1	7.4 ± 0.7	5.3 ± 1.6
TLC (×10^9^ cells/L)	2.63 ± 0.88	2.16 ± 0.65	2.88 ± 0.91	2.66 ± 0.69	2.58 ± 0.10	3.31 ± 0.35	2.64 ± 0.84
Platelets (×10^9^ cells/L)	82 ± 35	47 ± 20	84 ± 14	100 ± 23	70 ± 31	73 ± 19	82 ± 32
ANC (×10^9^ cells/L)	1.61 ± 0.82	0.79 ± 0.61	1.23 ± 0.95	1.66 ± 0.78	1.52 ± 0.65	2.22 ± 0.22	1.53 ± 0.83
RDW	16.9 ± 5.6	14.9 ± 2.3	15.0 ± 3.4	14.8 ± 4.8	14.8 ± 2.7	15.0 ± 2.0	16.1 ± 5.0
MCV (fl)	95.1 ± 14.7	90.0 ± 9.7	92.1 ± 7.1	85.9 ± 10.9	90.9 ± 3.3	94.8 ± 2.5	92.8 ± 13.1
MCH (pg)	27.7 ± 2.7	28.9 ± 3.8	27.5 ± 2.5	26.9 ± 2.9	27.3 ± 3.3	24.6 ± 0.9	27.5 ± 2.8
MCHC	33.5 ± 1.6	33.4 ± 2.3	32.6 ± 1.9	33.4 ± 1.2	33.6 ± 0.9	32.6 ± 1.2	33.4 ± 1.6
Retic count (%)	1.9 ± 0.9	0.7 ± 0.2	1.8 ± 0.8	1.5 ± 0.9	2.1 ± 0.8	1.5 ± 0.6	1.7 ± 0.9
Hypersegmented neutrophils	24 (30.4%)	0 (0%)	1 (9.1%)	1 (5%)	1 (25%)	0 (0%)	27 (21.3%)
Macrocytosis	29 (34.1%)	1 (9.0%)	1 (7.6%)	3 (13.04%)	1 (25%)	0 (0%)	35 (25%)
Macroovalocytosis	29 (34.1%)	1 (9.0%)	0 (0%)	2 (8.69%)	0 (0%)	0 (0%)	32 (21.2%)

Hb: hemoglobin; TLC: total leukocyte count; AEC: absolute eosinophil count; ANC: absolute neutrophil count; RDW: red cell distribution width; MCV: mean corpuscular volume; MCH: mean corpuscular hemoglobin; MCHC: mean corpuscular hemoglobin concentration; Retic count: reticulocyte count.

**Table 3:** Vitamin assays in pancytopenia.

	MA	AA	Leukemia	Infections	Myelo pthisis	Other	All Pancyto-penia
	Mean ± Standard deviation (SD)						
S. Ferritin (ng/mL)	38.3 ± 33.5	41.2 ± 33.4	55.6 ± 32.5	52.5 ± 40.4	57.7 ± 41.0	35 ± 29.2	42.9 ± 34.9
Vitamin B12 (pg/mL)	83.4 ± 59.2	220.3 ± 379.3	219.3 ± 536.1	155.5 ± 224.3	72.3 ± 26.6	1152.1 ± 2158.7	150.3 ± 426.8
Serum folate (ng/mL)	16.1 ± 4.5	15.7 ± 1.9	9.2 ± 4.7	8.8 ± 9.9	81.5 ± 109.7	57.4 ± 101.7	17 ± 4.3
S. Homo-cysteine (*μ*mol/L) *N* = 38	85.5 ± 33.4	68 ± 68	49.2 ± 48.0	48.8 ± 27.0	—	33 ± 1.41	73.6 ± 36.8
S. LDH (IU/L)	604.8 ± 618.2	278.2 ± 102.7	368.4 ± 112.1	194.3 ± 98	256.2 ± 87.4	401.5 ± 101.4	473.98 ± 764.8

MA: megaloblastic anemia; AA: aplastic anemia; LDH: lactate dehydrogenase.

**Table 4:** Hematological parameters of patients at follow up.

	MA	AA	Leukemia/lymphoma	Infections	Myelophthisis	Other	All Pancyto- penia	*P* value^†^
	*N* = 55	*N* = 8	*N* = 6	*N* = 12	*N* = 2	*N* = 3	*N* = 86	
		Mean ± standard deviation						
Hb (g/dL)	12.3 ± 0.9	9.4 ± 2.0	12.8 ± 0.9	12.3 ± 1.3	10.1 ± 4.6	11.1 ± 1.4	12.0 ± 1.5	**0.001**
TLC (×10^9^ cells/l)	7.5 ± 1.9	5.1 ± 2.1	8.2 ± 2.4	7.0± 2.6	6.6± 4.5	6.1± 1.4	7.2± 2.2	**0.001**
Platelets (×10^9^ cells/L)	267 ± 88	255 ± 138	256 ± 78	249 ± 69	180 ± 71	287 ± 142	262 ± 91	**0.001**
ANC (×10^9^ cells/L)	5.5 ± 1.5	3.5 ± 1.5	5.8 ± 1.7	4.8 ± 1.8	4.9 ± 4.1	4.3 ± 1.6	5.2 ± 1.7	**0.001**
RDW	12.8 ± 1.1	12.2 ± 0.6	13.4 ± 1.2	12.6 ± 1.1	12.8 ± 2.2	13.1 ± 1.7	12.7 ± 1.1	0.132
MCV (fl)	85.9 ± 6.7	86.6 ± 4.7	91.5 ± 4.9	86.1 ± 6.8	92.5 ± 2.4	88.3 ± 4.0	86.6 ± 6.4	**0.044**
MCHC	33.7 ± 1.7	32.5 ± 1.8	33.2 ± 1.7	34.0 ± 1.3	33.5 ± 1.2	32.8 ± 1.3	33.5 ± 1.6	0.476
MCH (pg)	29.6 ± 2.2	28.7 ± 2.4	29.4 ± 2.5	30.6 ± 3.1	31.2 ± 1.2	28.4 ± 3.8	29.7 ± 2.4	**0.001**
Retic count corrected	3.2 ± 1.1	1.7 ± 1.5	4.0 ± 1.4	2.6 ± 1.2	2.7 ± 0.7	3.5 ± 0.3	3.1 ± 1.3	**0.000**

Hb: hemoglobin; TLC: total leukocyte count; AEC: absolute eosinophil count; ANC: absolute neutrophil count; RDW: red cell distribution width; MCV: mean corpuscular volume; MCH: mean corpuscular hemoglobin; MCHC: mean corpuscular hemoglobin concentration; Retic count: reticulocyte count; LDH: lactate dehydrogenase.

^†^Student's *t*-test for significant difference of means at presentation and on follow up.

**Table 5:** Indian studies on pancytopenia.

S. No.	Study; location	Year	Age group (years)	No. of cases	Commonest cause of pancytopenia	2nd most common cause	Other causes
1	Varma and Dash; Chandigarh [9]	1992	Adults	202	AA (40%)	MA (23%)	AML, lymphoma.
2	Tilak and Jain; Chandigarh [12]	1999	5–70	77	MA (68%)	AA (7%)	Malaria, KZ, NHL, AML, MM, TB, Waldenstrom's macroglobulinemia
3	Khodke et al.; New Delhi [10]	2000	3–69	50	MA (44%)	AA (14%)	KZ, MM, HIV, MDS, AML, TB, Drug induced cytopenia
4	Kumar et al.; New Delhi [13]	2001	All ages	166	AA (29%)	MA (22%)	Aleukemic leukemia, lymphoma
5	Khunger et al.; New Delhi [14]	2002	2–70	250	MA (72%)	AA (14%)	Aleukemic leukemia, MDS, KZ, malaria, NHL, TB, myelofibrosis, MM
6	Santra and Das; Kolkata [11]	2010	13–65	111	AA (22%)	Hyper-splenism due to CLD (11%)	MA, SLE, Drug induced cytopenia, falciparum malaria, TB, CLL, MM, MDS, PNH, Felty's syndrome.
7	Present study; New Delhi	2008	18–80	140	MA (60%)	Leukemia (9%), AA (8%)	HIV, TB, KZ, Malaria, MDS, CML, Gaucher's disease, metastatic carcinoma, CLD with hypersplenism.

MA: megaloblastic anemia; AA: aplastic anemia; AML: acute myelogenous leukemia; KZ: kala-azar; MM: multiple myeloma; HIV: human immunodeficiency virus; MDS: myelodysplastic syndrome; TB: disseminated tuberculosis; NHL: non Hodgkin's lymphoma; CLL: chronic lymphatic leukemia; PNH: paroxysmal nocturnal hemoglobinuria; CML: chronic myelogenous leukemia; CLD: chronic liver disease.
